# HIV and STI Prevention Among Spanish Women Who have Sex with Women: Factors Associated with Dental Dam and Condom Use

**DOI:** 10.1007/s10461-022-03752-z

**Published:** 2022-07-05

**Authors:** María Dolores Gil-Llario, Vicente Morell-Mengual, Marta García-Barba, Juan E. Nebot-García, Rafael Ballester-Arnal

**Affiliations:** 1 Department of Developmental and Educational Psychology, University of Valencia, Valencia, Spain; 2 Department of Basic and Clinical Psychology and Psychobiology, Jaume I University, Castelló de la Plana, Castellon Spain

**Keywords:** HIV, Sexually transmitted infections, Lesbian, Dental dam, Condom use

## Abstract

The scientific community has systematically ignored the needs of women who have sex with women (WSW). The invisibilization of romantic and sexual relationships between women has caused a profound lack of knowledge about the impact of HIV and other STIs on this population subgroup. This study aims to analyze the frequency of dental dam and condom use in WSW and identify the variables that explain the use of these two preventive methods. The sample is composed of 327 women aged between 18 and 60 years (M = 27.82; SD = 8.10). The results indicate that only 4.7% of those who practice cunnilingus and 5.2% of those who practice anilingus report “always” using dental dam (systematic use). Condoms are used systematically by 37.1% of those who practice vaginal penetration and 37.8% of those who practice anal penetration. Age, high perceived self-efficacy, and adequate assertive communication skills are variables significantly related to preventive behavior. The severity attributed to HIV and the perceived risk of infection are protective factors regarding dental dam use. For condom use, high levels of internalized homophobia and drug use are risk factors. Future preventive strategies should provide information on preventive strategies to WSW who, for different reasons, are not involved in LGBT contexts or associations and, therefore, do not have access to training activities and mistakenly believe that they are invulnerable as they do not have sexual relations with men.

## Introduction

Women who have sex with women (WSW) constitute a heterogeneous subgroup that engages in a wide range of frequently unknown sexual practices [1, 2]. The social and institutional lack of visibility of these sexual relationships has led to a profound unawareness of the impact of HIV and other STIs on this group [3], excluding them from research and intervention policies [4–6]. The biased conception of sexuality from the perspective of heterosexual relations [7], together with the absence of epidemiological data and preventive programs, has fostered a false sense of invulnerability [8, 9]. However, the risk of contracting an STI is multifactorial and independent of sexual orientation, and anyone who disregards the use of barrier methods can become infected [10–12]. Transmission between women can occur through direct contact with menstrual blood, vaginal fluid exchange, or blood exposure from injuries suffered during sexual activity [13].

Currently, the incidence of some STIs among WSW is similar to the data reported in women who only have sex with men [14, 15]. Thus, Logie et al. indicate that one in five WSW has been diagnosed at least once with an STI [16]. The most reported STIs are human papillomavirus (HPV) and genital warts (30.6%), chlamydia (19.4%), oral or genital herpes (16.3%), and HIV (6.1%). The prevalence of STIs in women who only have sex with women ranges from 14.4 to 37.8% [2, 17–19], whereas, in women who have sex with men and women, the prevalence ranges between 7.4 and 37% at its lowest rates [2, 11, 19–21] and between 43.4 and 73.8% at its highest rates [17, 18, 22].

Particularly, HIV has been the STI that has received the least attention among WSW [23, 24]. This circumstance could increase the rates of late diagnosis, with repercussions on the likelihood of unknowingly transmitting the infection and developing AIDS due to not receiving the appropriate antiretroviral treatment [25]. Cases of HIV transmitted by sexual contact between women are difficult to establish since genetic analysis is required [13] and there are usually other associated risk factors that are considered more important, such as sex with men or intravenous drug use [26]. Other potential exposures associated with HIV transmission in WSW include use of shared sex toys and exposure to body fluids of others [13]. Furthermore, the estimation of prevalence and incidence figures is limited because epidemiological surveillance systems do not include a specific category for HIV or other STI transmission among WSW [12]. Nevertheless, there are some well-documented transmission cases of HIV between women in the scientific literature that demonstrate that absolute invulnerability is non-existent [13, 27]. A recent systematic review reported HIV prevalence rates ranging from 0 to 2.9% in Latin America and the Caribbean and between 7.7 and 9.6% in sub-Saharan Africa. Prevalence in other regions, such as Europe, was not reported [23].

The use of condoms and dental dams are very effective methods for the prevention of STIs and HIV. However, several studies indicate minimal use rates due to low-risk perception and the prevalence of misconceptions [20, 28]. Rowen et al. analyzed the use frequency of various preventive methods in a multiethnic sample of WSW [29]. The results indicate that only 6.1% of those who practice oral sex and 25.7% of those who practice genital stimulation with sex toys almost systematically use dental dams and condoms. This aspect is especially relevant because there is a documented case of HIV transmission between WSW using shared sex toys. The women reported having unprotected vaginal sex and using insertive sex toys that were shared between them but were not shared with any other persons [13]. Similar research conducted with Spanish WSW concludes that 3.1% of those practicing cunnilingus and 7.1% of those practicing anilingus with sporadic partners report systematic dental dam use [30].

Research that analyzes the variables that influence sexual risk behavior in WSW is very limited. The few existing studies conclude the importance of some cognitive-behavioral variables included in the explanatory models of behavior, such as, for example, knowledge level [20, 28], beliefs related to vulnerability and fear of contracting an STI [8, 9] and perceived self-efficacy [16]. On the other hand, the correlation between the use of barrier preventive methods and some effective, emotional, and behavioral factors has also aroused some interest in the scientific community. Thus, variables such as alcohol and other drug consumption [2, 22], sexual compulsiveness [30], the search for sexual sensations [31] and depressive symptomatology [19, 32], would constitute factors to be considered in developing preventive strategies. In addition, Logie et al. design, implement and evaluate the effectiveness of the only HIV and STI group prevention program for women who have sex with women [4]. The program is based on the Modified Social Ecological Model (MSEM) and includes behavioral and socio-structural variables related to knowledge, sexual assertiveness, self-efficacy, internalized homophobia, and self-esteem. The results of the programme show that self-efficacy, STI knowledge and sexual stigma are important variables to preventive behavior. However, all these variables have been scarcely studied and require a deeper analysis.

In recent years, there has been a gender approach to STI research, prevention, and treatment; however, this interest is focused almost exclusively on heterosexual women [33]. Preventive strategies that address the social and structural factors of vulnerability to HIV and other STIs in women who have sex with women are rarely studied properly [16]. Therefore, the objectives of this study are: to conduct a descriptive analysis of the frequency of dental dam and condom use; and to identify the combination of variables that best explain risky sexual behavior. The following hypotheses have been established:

1. Less than 5% of women with sporadic partners will report a systematic or always use of dental dam in oral sex.
2. Less than 25% of women with sporadic partners will report systematic or always use of condoms for vaginal or anal penetration.
3. The combination of the variables collected in the literature review will help explain why a high percentage of women do not use dental dams or condoms.

## Methods

### Participants

The sample is composed of 327 women who have sex with women between 18 and 60 years of age, with a mean age of 29 years (M = 27.82; SD = 9.35) and the most frequent ages ranging between 20 and 29 years (58.1%). The sample resides in different parts of Spain, with 27.5% living in the central-Eastern area, 18.7% in the Levante region, 17.1% in the North, 16.2% in the East, 12.8% in the South, and 8.6% in the central-Western area. According to educational level, 55.7% have university studies (52.9% have a bachelor’s or postgraduate degree and 2.8% have a doctorate), 42.8% have secondary studies, and 1.5% have primary studies. Regarding marital status, 66.1% reported being single, and 33.9% were in a stable relationship at the time of the evaluation. Table 1 shows the detailed data for both groups, women who have sex with women (WSW) and women who have sex with both women and men (WSWM).

**Table 1:** Sample characteristics

	Total (n = 327)	WSW (n = 183)	WSWM (n = 144)
Age			
Younger than 20 years old	8.9%	6%	12.5%
Between 20 and 29 years old	58.1%	52.5%	65.3%
Between 30 and 39 years old	23.1%	30.1%	16%
Between 40 and 49 years old	6.7%	8.7%	4.2%
Between 50 and 60 years old	2.4%	2.7%	2.1%
Residence area			
North region	17.1%	16.4%	18.1%
South region	12.2%	16.4%	6.9%
East region	34.9%	36.1%	33.4%
Central-Western region	10.4%	7.1%	14.5%
Central-Eastern region	25.4%	24%	27.1%
Educational level			
Primary schooling	1.5%	1.6%	1.4%
Secondary schooling	42.8%	38.3%	48.6%
Bachelor or Master Degree	52.9%	56.3%	48.6%
PhD	2.8%	3.8%	1.4%
Steady partner			
Yes	33.9%	32.2%	36.1%
No	66.1%	67.8%	63.9%

*WSW* women who have sex with women; *WSWM* women who have sex with women and men

### Instruments

#### Cognitive Variables

- CPS. AIDS Prevention Questionnaire [34]. This scale is a 44-item measure that assesses different determinants and factors related to HIV/AIDS prevention. They are divided into six components: knowledge about HIV, fear of HIV infection, perceived susceptibility, intentions of condom use, safe sexual behavior, condom attitudes, and stigma and discrimination towards people living with HIV. The items related to condom use are only limited to sexual relations with women. The internal consistency for the different factors ranged from 0.67 to 0.74
- LBSS. Latex Barrier use Self-efficacy Scale [35]. This instrument contains seven statements designed to evaluate different skills related to the use of the latex barrier at different moments in a sexual relationship. Responses are given on a five-point scale ranging from 1 «strongly disagree» to 5 «strongly agree», and thus possible scores range from 7 to 35 points, in which a higher score indicates high Self-efficacy. The original version used in this study has an internal consistency of 0.80.
- EBAP. Brief Condom Use Self-efficacy Scale [36]. This scale is a 7-item measure that assesses different skills related to the use of condoms like, fear of rejection (MR), impulse control (CI), and acquisition and negotiation (AN). The items related to condom use are only limited to sexual relations with women. It is answered by means of a 5-point scale ranging from 1 «completely disagree» to 5 «completely agree». The score ranges from 3 to 15 points to MR scale and from 2 to 10 points in CI and AN scales. Higher scores indicate high Self-efficacy to condom use. The original version used in this study has an internal consistency of 0.71.

#### Behavioral Variables

- Alcohol, cannabis and other drug use. Three self-rating items are used to assess the use of alcohol, cannabis or other drugs during sex in the past 6 months: Do you have sex after drinking alcohol?; Do you have sex after smoking cannabis?; and Do you have sex after using other drugs like cocaine, MDMA, GHB and amphetamines? Responses are recorded using a dichotomous format: yes or no.
- SCS. Sexual Compulsivity Scale [37, 38]. This instrument contains ten statements on a Likert-type scale ranging from 1 «not at all like me» to 4 «very much like me» to evaluate interference of sexual behavior and failure to control sexual impulses. The score ranges from 5 to 20 points in both scales. Higher scores indicate high sexual compulsivity. The Spanish adaption has an internal consistency of 0.84.
- SAS. Pregnancy and STD prevention assertiveness subscale [39, 40]. This subscale of the Sexual Assertiveness Scale specifically targets communication and negotiation skills regarding condom and latex barrier use. All items were formatted for a 5-point Likert-type response ranging from 0 «never» to 4 «always». Total score for the subscale ranges from 0 to 24 points, in which a higher score indicates high assertive communication skills. The internal consistency of the Spanish adaptation is 0.85.
- SSSS. Sexual Sensation Seeking Scale [31, 37]. This scale contains 11 statements to evaluate sexual sensation seeking behaviors and attitudes using a Likert-type scale ranging from 1 «Not at all like me» to 4 «Very much like me». The score ranges from 7 to 28 in Physical Sensations Attraction scale (PSA) and from 4 to 16 in New Experiences Seeking scale (NES). Internal consistency of Spanish version showed good reliability in each scale, ranging from 0.71 to 0.84.

#### Affective-Emotional Variables

- SIHS. Short Internalized Homonegativity Scale [41, 42]. This instrument contains 13 statements designed to evaluate social and sexual comfort with homosexual people and public identification as a homosexual using a Likert-type scale ranging from 1 «strongly agree» to 5 «strongly disagree». The score ranges from 4 to 20 in social (SOCC) and sexual comfort (SEXC) with homosexual people factors, and from 5 to 25 in public identification as homosexual factor (PIH). Higher scores indicate high homophobic attitudes. The Spanish version used has a good reliability in each factor, ranging from 0.70 to 0.78.
- RSES. Rosenberg Self-esteem Scale [43, 44]. It is a unidimensional instrument, made from a phenomenological conception of self, which measures the respect and acceptance of people to themselves. A 10-item scale whose items are answered using 4-point Likert scale format ranging from 1 «strongly agree» to 4 «strongly disagree». The total score ranges from 10 to 40 points, in which a higher score means high self-esteem. The internal consistency of the validated Spanish version is 0.85
- CES-D. Center for Epidemiological Studies of Depression Scale [45, 46]. It is a 7-item self-report brief instrument designed to identify various aspects related to depressive symptomatology, and employs 4-point Likert scales, ranging from 0 «rarely or none of the time» to 3 «most or all of the time». The total score ranges from 0 to 21 points, in which a higher score indicates more severe depressive symptoms. The Spanish brief version used has an internal consistency of 0.85.

### Procedure

Participants were recruited online. Firstly, several associations and NGOs were contacted (e.g., Spanish Federation of Lesbians, Gays, Transsexuals and Bisexuals, Youth Institute, student associations, among others) to provide information about the study and request their collaboration to disseminate the evaluation questionnaire. The associations that expressed their interest published a short text providing information about the study and a link to the evaluation platform through their websites or social networks (Facebook and Twitter). The first page provided information on the anonymity and confidentiality of participant data and collected their informed consent. This study followed the guidelines of the Declaration of Helsinki and the Spanish Personal Data Protection Law (Organic Law 15/1999). Participation was voluntary, and no financial or material remuneration was received.

### Analysis of Data

The article focuses on women who have sex with women, although some women also have sex with men (but these behaviors are not assessed in the study). Descriptive analyses were performed to examine the frequency of dental dam and condom use with “sporadic partners” (romantic relationships that are not completely exclusive between two people and where HIV status is unknown). Data from women who have a stable relationship (who are in a monogamous relationship and only have sex with one partner) are not included. According to the scientific literature, having a monogamous partner minimizes the risk of HIV infection or other STI. For this reason, prevention programmes should focus on people who have sex with multiple partners and do not know their HIV status. The chi-squared test was used as a contrast statistic to analyze the possible differences between WSW and WSWM. The effect size was calculated using Cramer’s V coefficient. Values of about 0.10 were considered small, those close to 0.30 were moderate, and those greater than 0.50, large. A logistic regression analysis was then carried out to identify the explanatory factors for using both preventive methods. Two dichotomous variables were generated from the items related to preventive behavior of the AIDS Prevention Questionnaire [34]. For dental dam use, a value of 0 was assigned to risk behavior, using dental dams ‘never’ or ‘sometimes’ and a value of 1 to preventive behavior, that is, using dental dams ‘often’ or ‘always’. For condom use, a value of 0 was assigned to risk behavior, in this case, not using a condom systematically or ‘never’, and a value of 1 to preventive behavior, which is systematic condom use.

## Results

### Description of Dental Dam and Condom Use Frequencies

The data collected reveal a high percentage of women who do not use preventive methods systematically or always (Table 2). The dental dam has a very low use frequency. Only 4.7% of women who practice cunnilingus and 5.2% of those practicing anilingus report systematic use in their relations with sporadic partners. The frequency of condom use is slightly higher. Accordingly, 37.1% of those who practice vaginal penetration and 37.8% of those who practice anal penetration report systematic use in their relations with sporadic partners. Finally, it should be noted that there are no statistically significant differences in the use frequency of the different preventive methods between women who only have sex with women and women who have sex with men and women.

**Table 2:** Dental dam and condom use frequencies in women with sporadic partners

	Total	WSW	WSWM	X^2^	df	p	V
Cunnilingus (dental dam)							
Never	79.9%	79.8%	80%	1.007	3	0.799	0.072
Sometimes	8.7%	8.4%	9.3%				
Usually	6.7%	7.6%	5.3%				
Always	4.7%	4.2%	5.3%				
Annilingus (dental dam)							
Never	81%	81.6%	80%	0.75	3	0.317	0.036
Sometimes	8.6%	7.9%	10%				
Usually	5.2%	5.3%	5%				
Always	5.2%	5.3%	5%				
Vaginal penetration (condom)							
Never	47.5%	47.9%	46.9%	4.363	3	0.225	0.175
Sometimes	9.1%	6.4%	14.4%				
Usually	6.3%	8.5%	2%				
Always	37.1%	37.2%	36.7%				
Anal penetration (condom)							
Never	52.3%	47.3%	63%	3.148	3	0.369	0.196
Sometimes	6.2%	5.5%	7.4%				
Usually	3.7%	5.5%	0%				
Always	37.8%	41.7%	29.6%				

*WSW* women who have sex with women; *WSWM* women who have sex with women and men

### Explanatory Variables of Dental Dam and Condom Use

A logistic regression analysis was carried out using the forward method (Wald) to identify which variables determine the use of the dental dam in oral sex (Table 3) and the use of condoms in vaginal or anal penetration (Table 4). The Hosmer and Lemeshow test results were adequate for both the model analyzing dental dam use (χ^2^_8_ = 2.410; p = 0.966) and the model analyzing condom use (χ^2^_8_ = 10.726; p = 0.218), indicating goodness of fit.

**Table 3:** Multiple regression logistic analysis of the dental dam use in oral sex in women with sporadic partners

	β	ET	Wald	gl	Sig	Exp (β)	IC 95% for (β)	
							Inferior	Superior
Age	0.082	0.027	8.872	1	0.003	1.085	1.028	1.145
Perceived severity HIV (CPS)	1.083	0.447	5.864	1	0.015	2.953	1.229	7.093
Perceived vulnerability to HIV (CPS)	0.032	0.013	6.041	1	0.014	1.032	1.006	1.059
Self-efficacy (LBSS)	0.124	0.044	8.054	1	0.005	1.131	1.039	1.232
Cannabis during sex	1.840	0.834	4.871	1	0.027	6.297	1.229	32.268
Sexual assertiveness (SAS)	0.098	0.044	5.059	1	0.024	1.103	1.013	1.201
Physical Sensations Attraction (SSSS)	− 0.155	0.078	3.925	1	0.048	0.856	0.734	0.998

**Table 4:** Multiple regression logistic analysis of the condom use in vaginal or anal penetration

	β	ET	Wald	gl	Sig	Exp (β)	IC 95% for (β)	
							Inferior	Superior
Age	0.095	0.030	9.997	1	0.002	1.100	1.037	1.167
Sexual orientation	− 1.211	0.511	5.602	1	0.018	0.298	0.109	0.812
Other drugs during sex	2.270	0.885	6.579	1	0.010	9.679	1.708	54.844
Sexual assertiveness (SAS)	0.153	0.045	11.597	1	0.001	1.165	1.067	1.272
Self-efficacy (EBAP)	0.097	0.048	4.068	1	0.044	1.102	1.003	1.210
social comfort with homosexual people (SOCC)	− 0.199	0.091	4.755	1	0.029	0.820	0.686	0.980

In women who engage in oral sex with sporadic partners (Table 3), an older age, perceiving HIV as a serious disease (CPS item), perceiving oneself as vulnerable to HIV infection (CPS item), high self-efficacy for the use of dental dam (LBSS score), not consuming cannabis before sexual intercourse, and good assertive communication skills (SAS score) are protective factors. In contrast, a high attraction to physical sensations (SSSS factor) is the only risk factor. Of all the variables analyzed, abstinence from cannabis use and the severity attributed to HIV multiply the probability of using dental dam by 6.297 and 2.953, respectively. Cox and Snell’s R^2^ (0.276) and Naglekerke’s R^2^ (0.427) values determine that the model explains between 27.6 and 42.7% of the variance in the dependent variable. Overall, a good classification result is obtained, with an average of 85.2% of the classifications performed correctly. The results are better for sensitivity since it correctly classifies 96.1% of the women who never use dental dams and slightly worse for specificity because it correctly classifies 45.2% of the women who use dental dams systematically or occasionally.

In women who practice vaginal or anal penetration with sex toys with sporadic partners (Table 4), having sex with both men and women (sexual orientation) and social discomfort with homosexuals (SOCC factor) increase the probability of not using condoms. On the other hand, older age, not using drugs before sexual intercourse, high self-efficacy for condom use (EBAP score) and good assertive communication skills (SAS score) are protective factors. Cox and Snell’s R^2^ (0.293) and Naglekerke’s (0.395) values determine that the proposed model explains between 29.3 and 39.5% of the variance. In addition, the model obtains an average of 75.5% of classifications performed correctly. The results are better for sensitivity, correctly classifying 83.5% of women who never use condoms, and lower for specificity, correctly classifying 63.8% of women who use condoms.

## Discussion

This study aimed to identify the percentage of women who have sex with women who use condoms and dental dams with sporadic partners and to analyze the combination of variables that explain this behavior. It is important to conduct this type of research for two reasons: on the one hand, the sexuality of this group has been a highly invisibilized aspect [2] and, on the other, the prevention of HIV and other STIs has been a problem that has been barely addressed by public bodies and the scientific community [7, 23].

The systematic frequency of dental dam use observed in this study is very low and confirms the hypothesis 1, although it is congruent with the figures reported in other studies with rates ranging from 2.3 to 7% [26, 29, 30]. However, other studies present slightly higher percentages of up to 11.2% [47]. These discrepancies could be due to the cultural context of the population studied or the time period to which their use is circumscribed (last 3 months, last 6 months) [26, 30]. On the one hand, the authorities responsible for epidemiological surveillance do not provide incidence and prevalence data [12]. Statistics on this group are not included or are grouped into general categories that make it difficult to collect reliable data. On the other hand, there are few preventive programs designed for WSW that include information adapted to their psychosocial reality [4]. Most preventive campaigns have focused on men who have sex with men, heterosexuals and intravenous drug users [48]. The lack of data leads to a false sense of invulnerability, which, in turn, is enhanced by a lack of knowledge of existing preventive methods. Fishman and Anderson indicate that 19.1% do not know how to make a dental dam from a condom [49].

Logistic regression analysis shows that age, self-efficacy and sexual assertiveness are the main explanatory variables for preventive behavior. So, hypothesis 3 is confirmed. Older age is a protective factor. Young WSW have a biased sexual education based on heterosexual patterns [5], since public organizations have not implemented specific programs [50]. Accordingly, Fishman & Anderson indicate that only 6% of lesbian women have received sexual information from public organizations [49]. It seems logical to assume that only when these women are older and go to associations or LGTB environments, where specific educational programs are implemented, will they have the possibility of acquiring knowledge adapted to their reality and their needs. Overall, WSW who use preventive methods feel empowered to use them in the face of extrinsic and intrinsic impediments [4]. One situation requiring a higher level of self-efficacy is associated with a possible negative evaluation when the use of the dental dam is suggested. Since it is a method that is not widely used, some women believe that its use is only circumscribed when there is a medical reason to justify it [51]. These women also possess the necessary assertive communication skills to initiate a sexual interaction, propose and negotiate the use of condoms and dental dams, and reject any unwanted sexual activity [16]. As indicated by Nesoff et al. these skills are necessary to manage any sexual interaction to occur as desired [52].

In using dental dams, the perceived severity of HIV and the perceived risk of infection are protective factors. Low perception of risk and severity decreases the probability of adopting preventive behaviors [8]. These variables are a social construct influenced by the emergence of the erroneously named risk groups. Seeing oneself outside these groups can create a false sense of invulnerability to HIV and other STIs [9]. According to Dolan & Davis it is likely that women who report not using dental dams believe that if a woman looks healthy, she is healthy, and no additional preventive measures are necessary [53]. In addition, as Fujii, concludes, not consuming cannabis before sexual intercourse is another protective factor [2]. Being under the depressant effects of cannabis causes an impairment in the decision-making process [54], leading to an underestimation of risk and a greater likelihood of not using dental dam [22]. Finally, the search for sexual sensations appears as a risk variable. This finding is congruent with the few studies that have analyzed this variable [31]. These women generally think that physical sensations are the most important aspect of sex and prefer the short-term benefits of sexual practices that disregard the use of barrier methods [54, 55]. Therefore, the greater discomfort caused by the use of the dental dam or the interruption that its use generates in the sexual dynamic are aspects that can diminish physical sensations or sexual satisfaction and, consequently, may drastically reduce its use [56].

Sexual orientation and internalized homophobia are risk factors in condom use. Women who have sex with women and men are more likely to disregard the use of condoms. In general, these women have received a heterocentric affective-sexual education that only considers the use of condoms in sexual relations with men [5, 7]. When they have sex with women, they have a false feeling of invulnerability and therefore choose not to use this preventive method [9]. The results of the present study indicate that a higher level of internalized homophobia, related to social comfort when in settings frequented by openly homosexual people decreases condom use. This finding is consistent with other studies that analyze this variable [4, 16]. The prejudices associated with homosexuality are so widespread that even homosexual and bisexual people end up internalizing and accepting them, even if they go against themselves [57, 58]. It is likely that women with high internalized homophobia are not integrated within the LGBT community and, therefore, do not regularly attend places where preventive programs are implemented [4]. For their part, not consuming drugs other than alcohol or cannabis is a protective factor. These women tend to consume some narcotic substances, such as cocaine or hallucinogens, to a greater extent than the general population [59]. The behavioral effects of these substances include impaired judgment or decision-making, which leads to an increased likelihood of engaging in risky behaviors [54].

This study has some limitations. The sample were exclusively recruited online through LGBT Spanish organizations, and thus results cannot be generalized to WSW who are not comfortable engaging in LGBT organizations. Knowledge questions were limited to condom and latex barrier use. Future studies should include questions on other preventive methods, like sexual negotiation skills, STI/HIV tests before intercourse, Pre-exposure prophylaxis (PrEP) and Post-exposure Prophylaxis (PEP). In addition, because of the cross-sectional design, the causal relationships among the variables should be interpreted with caution. In future studies, it would be advisable to design a longitudinal research to confirm the causality of the current findings. Finally, the assessment of frequency about condom and dental dam use was general, not at last sex. This type of evaluation could hinder recall.

## Conclusions, Contribution and Practical Implications

The scientific community and health professionals have systematically ignored the sexual health of women who have sex with women. This circumstance has led to a certain lack of knowledge about the impact of STIs and HIV on this group. There is now evidence that the risk of HIV and other STIs is multidisciplinary and independent of gender or sexual orientation. Anyone who has an active sex life and does not use condoms or dental dams has some degree of risk to a certain extent. This circumstance evidences the need to understand the factors that lead to risky sexual behavior in WSW to design preventive strategies based on scientific evidence. In this sense, the contribution of this study is that it proposes a model integrated by several cognitive-behavioral factors that explain the use of dental dam and condoms in Spanish women who have sporadic relationships with women. The model establishes that being older, perceiving a high level of self-efficacy, and possessing adequate assertive communication skills are variables strongly linked to the practice of preventive behaviors. Specifically, perceived HIV severity and risk and not consuming cannabis are variables that increase the use of dental dams. On the other hand, high levels of internalized homophobia and drug use are factors that decrease condom use. These results have significant practical implications for the design of preventive strategies. Specifically, information on preventive methods adapted to the psychosexual reality of this group should be provided. It is also advisable to improve the beliefs that each person has about their ability to adopt preventive behaviors and provide training in assertive communication skills that allow them to suggest and negotiate the most appropriate preventive method for each sexual practice. As specific aspects, risk reduction strategies associated with the use of cannabis or other drugs should be implemented and tools to reduce negative beliefs towards homosexuality and analyze the possible negative consequences derived from risky behaviors.

## Data Availability

Not applicable.

## Code Availability

Not Applicability.
